# Adsorption based realistic molecular model of amorphous kerogen

**DOI:** 10.1039/d0ra04453a

**Published:** 2020-06-18

**Authors:** Hyeonseok Lee, Farnaz A. Shakib, Kouqi Liu, Bo Liu, Bailey Bubach, Rajender S. Varma, Ho Won Jang, Mohammadreza Shokouhimher, Mehdi Ostadhassan

**Affiliations:** Key Laboratory of Continental Shale Hydrocarbon Accumulation and Efficient Development, Ministry of Education, Northeast Petroleum University Daqing 163318 China mehdi.ostadhassan@nepu.edu.cn; Department of Petroleum Engineering, University of North Dakota Grand Forks ND 58202 USA; Department of Chemistry and Environmental Science, New Jersey Institute of Technology Newark New Jersey 07102 USA shakib@njit.edu; Department of Materials Science and Engineering, Research Institute of Advanced Materials, Seoul National University Seoul 08826 Republic of Korea hwjang@snu.ac.kr mrsh2@snu.ac.kr; Regional Centre of Advanced Technologies and Materials, Faculty of Science, Palacky University Šlechtitelů 27 783 71 Olomouc Czech Republic

## Abstract

This paper reports the results of Grand Canonical Monte Carlo (GCMC)/molecular dynamics (MD) simulations of N_2_ and CO_2_ gas adsorption on three different organic geomacromolecule (kerogen) models. Molecular models of kerogen, although being continuously developed through various analytical and theoretical methods, still require further research due to the complexity and variability of the organic matter. In this joint theory and experiment study, three different kerogen models, with varying chemical compositions and structure from the Bakken, were constructed based on the acquired analytic data by Kelemen *et al.* in 2007: ^13^C nuclear magnetic resonance (^13^C-NMR), X-ray photoelectron spectroscopy (XPS), and X-ray absorption near-edge structure (XANES). N_2_ and CO_2_ gas adsorption isotherms obtained from GCMC/MD simulations are in very good agreement with the experimental isotherms of physical samples that had a similar geochemical composition and thermal maturity. The N_2_/CO_2_ uptake by the kerogen model at a range of pressure shows considerable similarity with our experimental data. The stronger interaction of CO_2_ molecules with the model leads to the penetration of CO_2_ molecules to the sub-surface levels in contrast to N_2_ molecules being concentrated on the surface of kerogen. These results suggest the important role of kerogen in the separation and transport of gas in organic-rich shale that are the target for sequestration of CO_2_ and/or enhanced oil recovery (EOR).

## Abstract 2

This paper reports the results of Grand Canonical Monte Carlo (GCMC)/molecular dynamics (MD) simulations of N_2_ and CO_2_ gas adsorption on three different organic geomacromolecule (kerogen) models.

## Graphical abstract

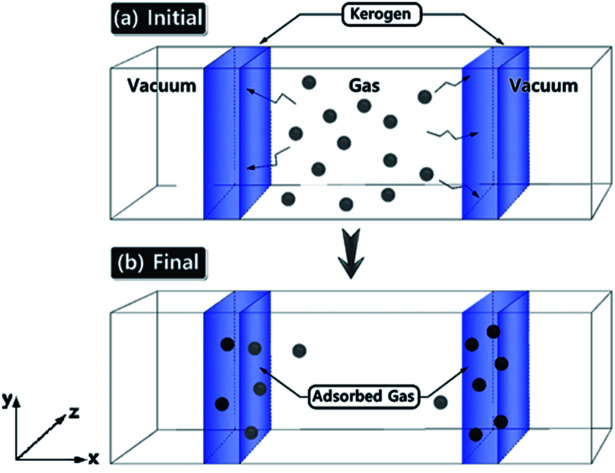

## Introduction

The worldwide increase of energy consumption was accompanied by a shift of interest from conventional resources to the unconventional shale gas and oil^1^ leading to continuous research and development on how to extract from these reservoirs^2,3^ even though such reservoirs require more costly and advanced technologies to exploit.^4–6^ In these reservoirs, organic matter or kerogen, which is the source of hydrocarbons,^7,8^ is a major but poorly understood constituent compared to inorganic minerals. This is mostly because of the complexity in chemical composition, structure, and properties of kerogen which originates from its biogenic origin.^9^ Kerogen, composed of mainly carbon, hydrogen, oxygen, nitrogen, and sulfur, experiences major structural and compositional changes as it undergoes maturation as a function of burial depth, *i.e.* pressure and temperature,^10^ and finally breaks down to petroleum and other by-products. Maturation is a complex chemical transformation that encompasses free-radical mechanisms, causing the investigation of volumetric, thermodynamic, and stereochemical properties of porous kerogen to be a highly taxing process. Furthermore, the high submicron porosity of kerogen drastically impacts the storage and transport properties of the entire shale layer^1,7^ and adds another layer of complexity to the investigation of this macromolecule. Therefore, building molecular models for kerogen is a much desired but challenging task, and not surprisingly, it has been continuously evolving with the advancements in computational methods.^8,11^

The first kerogen model was published by Burlingame *et al.* in 1968 which had focused on the study of the kerogen extracted from the Green River shale.^12^ The suggested model did not represent a comprehensive chemical structure of the sample though, as it did not contain molecular topology. Later in 1995, Siskin *et al.* proposed an updated model for kerogen, particularly by adding the functional groups with oxygen and nitrogen.^13^ Recent advancements in computational 3D modeling, drastically renewed the interest in exploring kerogen's molecular structure. Varying types of maturity models were introduced for kerogen by Ungerer *et al.* in 2015 (ref. 14) wherein they analyzed diverse kerogen types (based on their biogenic origin) grounded on a set of experimental data and PM7 semiempirical calculations as implemented in MOPAC.^15^ In addition to the development of the molecular models for this purpose, the computational techniques have also become frequent tools for simulating the gas adsorption and desorption processes.^16–18^ Simulation of adsorption behavior is important since organic-rich shales are becoming a repository of greenhouse gas storage which can also improve their productivity in CO_2_ enhanced oil recovery (EOR) and sequestration.

The Bakken formation is one of the largest unconventional shale oil plays in North America and is currently being studied for potential CO_2_-enhanced oil recovery and sequestration;^19^ recent studies suggest that an injection of CO_2_ into organic-rich shales can increase their production potential.^16,19^ Hence, in order to precisely estimate the capacity of organic matter in terms of adsorption for sequestration and/or associated mechanisms for enhanced oil recovery, building a 3D molecular model of the Bakken kerogen has become imperative. Here, we report a new representative molecular model for organic matter from the Bakken (kerogen type II) based on previous experimental chemical compositional data.^20^ We validate our models with gas (CO_2_ and N_2_) adsorption isotherms based on both experimental techniques and theoretical simulations. We also investigate CO_2_ and N_2_ diffusion behavior in the kerogen system to present a complete picture of interactions that would occur between kerogen and gas molecules.

## Methods

### Model preparation

A variety of methods can be utilized to provide the chemical composition of organic matter. While ^13^C-NMR is used to examine the chemical structures and parameters related to carbon, the sulfur and nitrogen content are revealed through the X-ray absorption near-edge structure (XANES) analysis. The X-ray photoelectron spectroscopy (XPS) is capable of quantifying several functional groups in carbonaceous materials associated with carbon, oxygen, sulfur, and nitrogen.^21^ This information can then be used to build a representative model of any organic material. Here, to build our molecular model of Bakken kerogen, we use the chemical and structural information of kerogen from diverse origins including Bakken, using ^13^C-NMR, XPS, and XANES data as were reported by Kelemen *et al.* in 2007.^20^ The Bakken organic matter is an immature (pre-oil window) type II kerogen representing a marine environment with *T*_max_ of 419 °C and hydrogen index (HI) of 580 mg g^−1^.^20^ The atomic ratios of carbon, hydrogen, sulfur, and nitrogen atoms were decided by considering the ^13^C-NMR, XANES, and XPS analysis results. In particular, ^13^C-NMR data were utilized for carbon and XPS/XANES for heteroatoms estimations to build the molecular model. From this data, around 35% of the total carbon concentration is included within aromatic structures that also contain nitrogen and sulfur, such as pyridine, pyrrole, and thiophene. The functional groups related to sulfur were set as sulfate and sulfoxide structures, while the oxygen-related functional groups were set as carbonyl and ether.

Throughout the text, the theoretical results obtained from our molecular model of the Bakken kerogen will be compared and contrasted to a set of experimental results from literature^20^ plus two other sets of results that were collected from the Bakken (type II) and tested for gas adsorption in our lab. For the ease of comparison, we refer to the first set of experimental results as sample B1 and the other two as samples B2 and B3 (sampled at 8387 and 9814 feet in vertical depths, respectively). The geochemical characteristics of all these three samples, obtained from programmed pyrolysis,^22^ are reported in Table 1. It can be seen from this table that the two B2 and B3 samples have the same *T*_max_ of 429 °C, and hydrogen index (HI) of 555 and 513 mg g^−1^, respectively. Based on this analysis, we conclude that all of these three samples have similar chemical and physical properties and can equivalently represent the immature Bakken kerogen since they are all in the pre-oil generation window. Thus, while we used the data from sample B1 for building molecular models, we obtained the adsorption isotherm data from sample B2 and B3 to verify proposed molecular model.

**Table 1:** Properties of the Bakken shale kerogen samples, all belonging to type II kerogen and in the pre-oil window (immature)

Property	B1^a^	B2^b^	B3^b^
*T*_max_ (°C)	419	429	429
HI (mg g^−1^)	580	555	513

a *T*_max_ and HI data of Bakken sample B1 were estimated by Kelemen *et al.*^20^

b For Bakken sample B2 and B3, Rock-Eval pyrolysis was applied to quantify *T*_max_ and HI.^22^

### Molecular model building

The construction of macromolecule kerogen models in this paper consists of the following major steps.

(a) First, the details of chemical composition including the nature and ratio of functional groups were determined through analyzing the experimental data reported by Kelemen *et al.*,^20^ sample B1.

(b) Using this information, fragments of monoaromatic/polyaromatics moieties (benzene, pyrrolic, pyridinic, and thiophene) and functional groups (sulfate, sulfoxide, carboxylate, amino) and alkanes were built using molecular drawing software, Avogadro.^23^ These fragments were built using General AMBER Force Field (GAFF) parameters.^24,25^ The fragments contained the as accurate number of nitrogen, sulfur, and aromatic carbon atoms as possible based on the experimental data. The partial charges on all atoms were assigned by the Gasteiger–Marsili sigma charges^26^ at the initial stage of the macromolecular model building. The nature of bridges (*e.g.* ketone and ether) between the fragments were assigned based on the sample analysis and were selected to satisfy the number of oxygen, and carbon atoms.

(c) In designing aromatic fragments, ^13^C-NMR data were used to find the percentage of protonated, non-protonated, and bridge carbons, where XPS results were used to obtain the ratio of nitrogen and sulfur-containing aromatic structure.

(d) In order to cross-link all of the prebuilt fragments, we used the “bond creation” feature of the LAMMPS package.^27^ This feature can create bonds between specified atomic sites as a molecular dynamics (MD) simulation running, if the distance between the two atoms becomes less than a threshold value. As such, we carefully selected the bonding sites in the form of aromatic carbon (protonated, non-protonated) and oxygen-related fragments, because in that format they can better fit the designed model. The pre-built fragments were positioned in a rectangular simulation box using Packmol package.^28^ Then, the cross-linkings between the fragments and bridges were generated during an MD trajectory that converged towards local equilibrium with GAFF force field parameters.^25,27^

(e) When the fragments were branched, conforming to the desired ratio of hydrogen to carbon atoms led to the creation of unpaired free radical sites. Therefore, the cross-linked fragments were inspected and improved maximally by adding or removing hydrogen or methyl groups. Thus, by trial and error process, we built the molecular model of kerogen that interweaves all of the constituent fragments within a single macromolecule.

### Quantum mechanics calculations

To obtain the quantitative electrostatic properties and optimized geometries of our kerogen model, we performed quantum mechanical (QM) calculations using the ORCA package^29^ based on the density functional theory (DFT) method. As DFT considered to be suitable for organic compounds, we ran our calculations at B3LYP/6-31G(d) method/basis set level.^30^ Hirshfeld atomic population^31^ analysis was carried out to obtain atomic partial charges since it is less basis-set dependent and can be derived for optimal partitioning of electron density. The partial charges obtained from the QM calculations replaced the initial partial charges which had been set without the polarization of atoms.

### Gas adsorption and diffusion simulation (GCMC + MD)

**Fig. 1:**
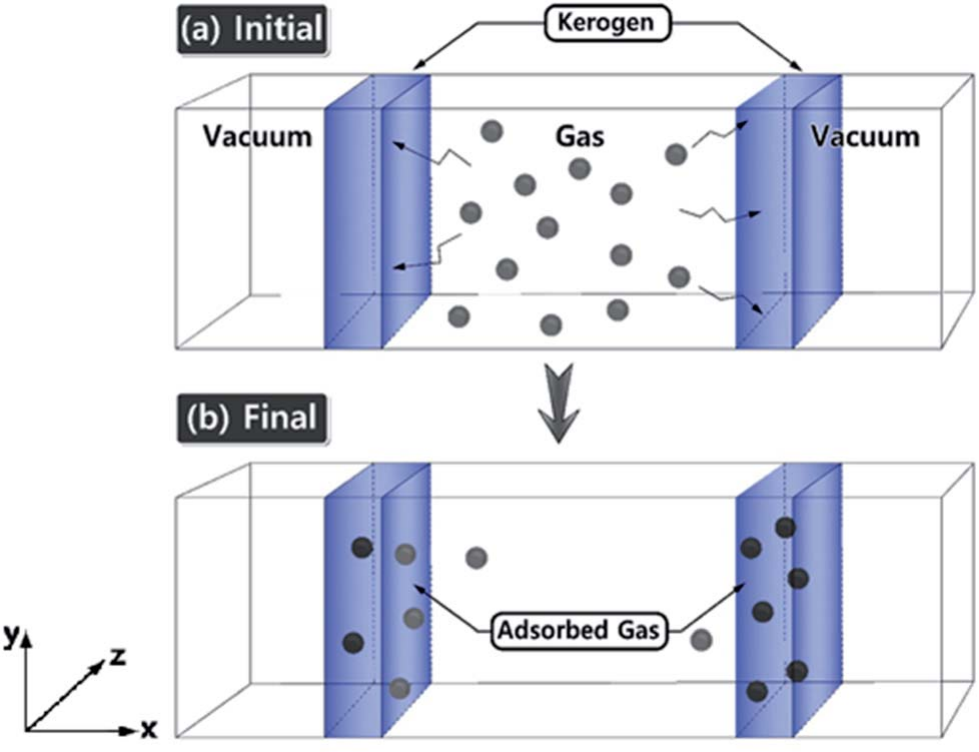
Schematic illustration of the simulation system. (a) Initial gas adsorption/diffusion simulation set-up within the kerogen models. (b) The system becomes equilibrated and the gas molecules are diffused. Gas molecules are diffuse and adsorbed by driving force along the *x*-axis.

Gas adsorption simulations were carried out using Grand Canonical Monte Carlo (GCMC) simulation, and gas diffusion simulations were utilized by Molecular Dynamics (MD) technique efficiently converging towards local equilibrium for diffusion equation. The equilibrium can be determined in the molecule configuration considering fluctuations in the internal energy and number of adsorbed molecules. We used a hybrid molecular simulation that consists of combining GCMC and MD to perform simulations implemented in LAMMPS package^27,32^ (schematic illustration of the simulation system is shown in Fig. 1). At every time step of the simulation, we attempted both GCMC exchanges (insertions and deletions) and MC moves (translations and rotations), followed by MD simulation steps in the canonical ensemble at the constant number of molecules. This process allows the gas molecule diffusion and kerogen model relaxation at each GCMC time step. Technically, every 100 GCMC insertion/deletion attempts followed by 200 MD time steps. In GCMC simulation, the chemical potential of the gas phase was related to the gas pressure using the ideal gas equation of state. The Metropolis algorithm was utilized to calculate the potential energy in the system and to control GCMC exchange or MC move. The gas adsorption and diffusion simulations were run for 5 × 10^7^ MD steps and 2.5 × 10^5^ GCMC cycles using a Nosé–Hoover thermostat to keep the temperature constant. The time step in all simulations was 1 fs.

Interactions were modeled by the sum of short-range Pauli repulsion and long-range electrostatic attraction embedded within Lennard-Jones potential with a cutoff distance of 10 Å using a particle–particle particle–mesh solver (PPPM).^33,34^ The N_2_ and CO_2_ molecules were simulated using the TraPPE force field parameter set shown in Table 2,^35^ which is useful for complex chemical systems with molecular simulation. In the TraPPE force field, CO_2_ was modeled as a linear triatomic and N_2_ as a diatomic molecule with fixed bond lengths and bond angles. These models are suitable for reproducing the densities and the diffusion of N_2_ and CO_2_ in bulk and surface phases at the conditions simulated in this work. The system was set in order to maintain a constant temperature of 77 K and 273 K which is the experimental gas adsorption temperatures and applied with the Nosé–Hoover thermostats. All partial charges of the kerogen models were obtained from QM calculations as explained in the previous section. At equilibrium, the number of gas molecules in the kerogen surface and bulk phase was kept constant.

**Table 2:** Parameters related to the adsorbates (CO_2_ and N_2_).^35^

Molecules	Atoms	Charge	*σ* (Å)	*ε*/*k*_B_ (K)
N_2_	N	−0.482	3.31	36.0
	N-COM	+0.964		0.0
CO_2_	C	+0.70	2.80	27.0
	O	−0.35	3.05	79.0

### Gas adsorption experiment

Gas adsorption experiments were performed on isolated kerogen from the bulk shale based on already established procedures.^36^ Briefly, we collected the samples and removed the bitumen using a mixture of methanol and toluene. Then, we added HCl into the solid residue to remove carbonates. Subsequently, HF was added to remove the silicate minerals, and pyrite was removed by using CrCl_2_, and finally, acid with dissolved inorganic minerals was separated from the organic matter by centrifugation.

After isolation from the rock matrix, the solid kerogen was degassed for at least 8 hours at 110 °C to remove moisture and volatiles, crushed (to less than 250 μm size) and loaded into the instruments. Low-pressure N_2_ was measured on a Micromeritics® Tristar II apparatus at 77 K while CO_2_ adsorption was performed on a Micromeritics® Tristar II plus apparatus at 273 K. The gas adsorption quantity was measured over the relative equilibrium adsorption pressure (*P*/*P*_0_) range of 0.01–0.99, where *P* is the gas vapor pressure in the system and *P*_0_ is the saturation pressure of N_2_.

## Results and discussion

### Bakken molecular models

**Fig. 2:**
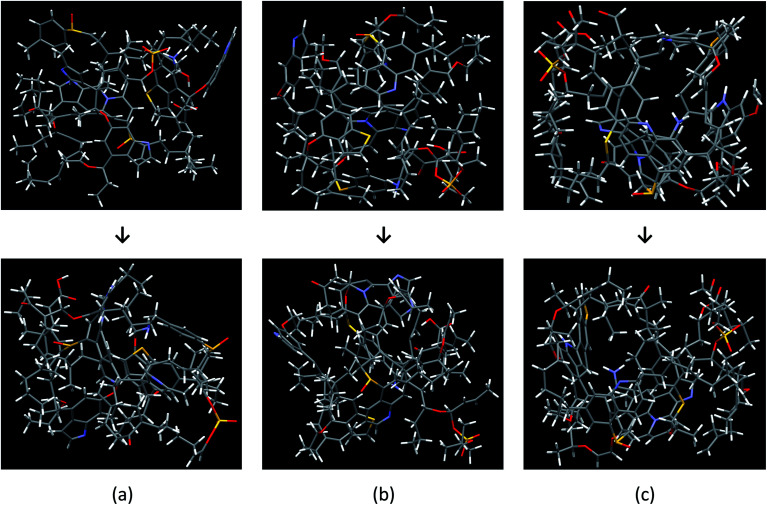
Constructed and optimized Bakken kerogen model A (a), model B (b), and model C (c). Different geometric configuration and chemical compositions with the following color code, carbon: black; hydrogen: white; oxygen: red; sulfur: yellow and nitrogen: blue. (a) C_141_H_187_N_6_O_15_S_4_, (b) C_152_H_193_N_6_O_15_S_4_, and (c) C_158_H_207_N_6_O_16_S_4_.

The Bakken shale models were constructed and verified by analyzing experimental data coupled with computational techniques (molecular builder, quantum mechanics calculations, and Monte Carlo/molecular dynamics simulation). The models consist of a complicated mixture of chain and mesh structures. Fig. 2 visualizes the three molecular models, before and after the optimization process, which do not have the same chemical composition and structure. The final chemical compositions of models A, B, and C are C_141_H_187_N_6_O_15_S_4_, C_152_H_193_N_6_O_15_S_4_, and C_158_H_207_N_6_O_16_S_4_, respectively.

Table 3 summarizes the aromatic carbons in the constructed models that were found compatible with ^13^C-NMR data in sample B1. Since aromatic carbons were set up at the initial stage of molecular model building, where aromatic fragments were prebuilt, carbons in aromatic structures are very close to sample B1 in regard to the structural parameters (*e.g.* protonated, non-protonated, and bridged carbon in aromatic structure). However, some discrepancies were detected such as the ratio of hydrogen to carbon atoms and methylene/methine structure. Because we improved the models by adding or removing methyl groups and hydrogens, it was not possible for every structure parameter of the models to meet the sample B1 perfectly.

**Table 3:** Structural parameters relevant to carbons in the Bakken kerogen (sample B1 (ref. 20)) and the constructed models (A, B, and C)^a^

Structure	Sample B1	Model A	Model B	Model C
Aromatic	0.35	0.371	0.344	0.330
Carboxyl/amide/carbonyl	0.02	0.028	0.026	0.025
Protonated aromatic	0.17	0.180	0.180	0.140
Phenoxy/phenolic	0.02	0.021	0.021	0.021
Alkyl-substituted aromatic	0.08	0.064	0.064	0.070
Bridged aromatic	0.09	0.092	0.092	0.092
Aliphatic	0.63	0.61	0.63	0.64
Methylene/methine	0.46	0.44	0.45	0.48
Methyl/methoxy	0.15	0.12	0.12	0.13
Alcohol/ether	0.06	0.04	0.04	0.04
H/C ratio	1.22	1.32	1.27	1.31
Average density (g cm^−3^)	—	0.927	0.919	0.974

a The data presented here are ratio per 1 number of carbon.

**Fig. 3:**
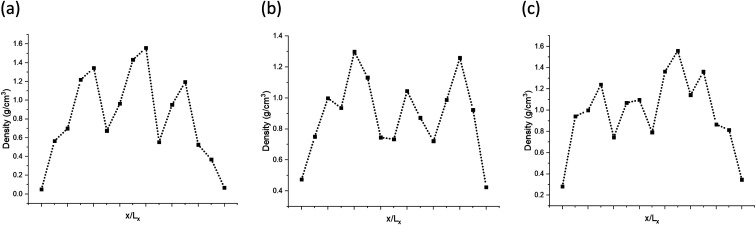
Density (g cm^−3^) profiles of the model A (a), B (b), and C (c) along the *x*-axis.

The models have average densities between 0.92 and 0.98 g cm^−3^ (in Table 3) demonstrating density profiles along the *x*-axis around 1.6 to 0.1 g cm^−3^ (in Fig. 3). The density profiles of models exhibit that the generated kerogen structures are amorphous and the internal/external surfaces are rough at the sub-nanometer level in Fig. 3. Since gas molecules diffuse and adsorbed along the *x*-axis (Fig. 1), the gas molecules could heavily be affected this internal density of models.

**Fig. 4:**
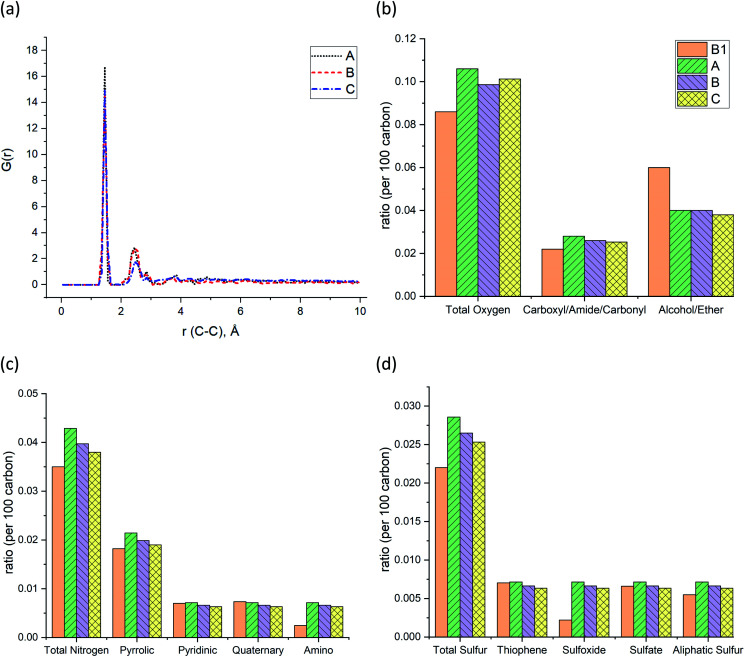
(a) Pair distribution functions (PDF) or G(r) of the Bakken kerogen models. The comparison between sample B1 and the models in terms of (b) the ratio of oxygen-containing functional groups, (c) the ratio of nitrogen-containing functional groups, and (d) the ratio of sulfur-containing functional groups.

The pair distribution function (PDF) profile, Fig. 4a, shows the probability of carbon existence at the distance *r* (Å) from another carbon, and it is exclusively related to carbon structure. The highest peak position is between 1.4 and 1.45 Å which represents aromatic carbons. Since almost 35% of the carbons in the models have an aromatic structure, this is the highest peak of all. The three models have similar peak positions with a similar width. Fig. 4b shows the comparison of the three models with sample B1 based on the total oxygen, *i.e.* carbonyl, ether, and alcohol groups per 100 carbons. It is apparent from this figure that the constructed models (A–C) contain a higher total number of oxygens per carbon than sample B1. It is the result of sample B1 having a higher number of ether and alcohol groups compared to the models but a similar ratio of carbonyl functional groups. It should be noted since the carboxyl and alcohol groups both contain –OH, the peak involving ether and alcohol groups could have been overlapped and intensified as a result, in the XPS spectrum.^20^ This phenomenon has led to a higher amount of alcohol/ether as a result of the summation of carboxyl and alcohol with –OH group shown in Fig. 4b. Fig. 4c and d also indicate that both prebuilt aromatic fragments (pyrrolic, pyridinic, and thiophene) and the bridges (quaternary, sulfate, and aliphatic sulfur) have comparable ratio with sample B1. However, the ratio of amino and sulfoxide in the models are somewhat higher. Screening Fig. 4b–d, one can also find that all three models have a smaller percentage of oxygen, nitrogen, and sulfur atoms than the original input data from XPS of sample B1. Our macromolecule models of Bakken kerogen consist of around 150 carbon atoms due to the size limitation of the model building. This limited total number of atoms in the models is not enough to thoroughly represent the perfect ratios.

### Gas diffusion/adsorption on the surface of kerogen

The ultimate goal of this study is to develop reliable amorphous kerogen molecular models. In order to verify the reliability of these models, here we compare and contrast the gas adsorption/diffusion simulation results of these models with the experimental gas adsorption isotherms that we have obtained from Bakken kerogen samples B2 and B3. We performed GCMC/MD simulations to investigate N_2_ and CO_2_ gas molecular adsorption on the surface of the models as well as their diffusion to the sub-surface levels. Apart from validating our models, because the three kerogen models cover a variety of structure and chemical composition and contain small size pore (<1 nm) that are irregularly spread all over the models, we expect that this study sufficiently clarifies the behavior of N_2_ and CO_2_ molecules through the small size pores of organic matter.

**Fig. 5:**
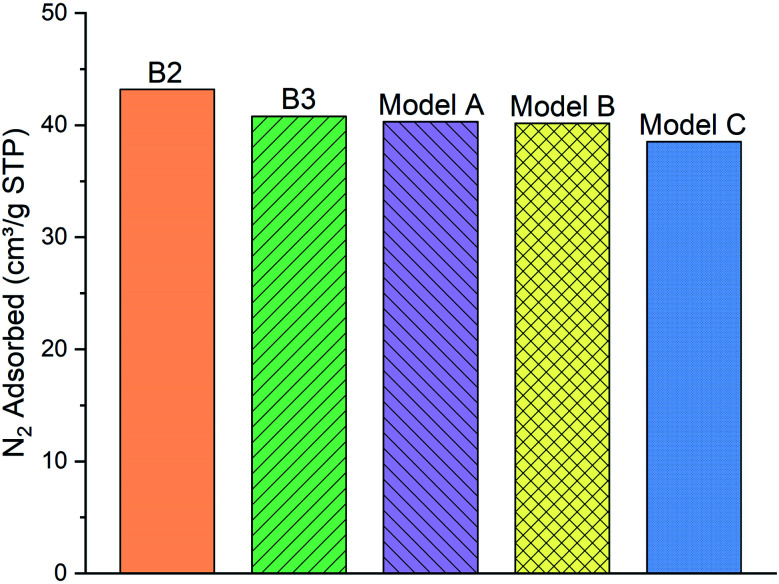
The comparison of the simulated excess nitrogen (N_2_) adsorption isotherms between the models (A–C) with experimental loadings (sample B2 and B3) at 100 kPa, 77 K.

First, we focus on the results of adsorption/diffusion of N_2_ molecules on/into three molecular models (A–C) and compare the results to the experimental N_2_ adsorption isotherms of samples B2 and B3 in Fig. 5.

As can be clearly seen, samples B2 and B3 capture around 43.18 and 40.78 (cm^3^ g^−1^ STP) of the N_2_ gas, respectively, at 100 kPa and 77 K. N_2_ molecules adsorption behavior with the model A and B (40.29 and 40.15 cm^3^ g^−1^ STP, respectively) are fairly close to the two experimental samples. Meanwhile, the number of adsorbed molecules into model C (38.5 cm^3^ g^−1^ STP) is almost 4% lower than both of the other two models and the experimental samples B2 and B3. We conclude that the difference of functional group distribution and internal density profile among the three models affects the adsorption of N_2_ molecules on the kerogen surface and pores. For instance, the model C containing a larger ratio of aliphatic carbon structure, specifically more methyl groups, cannot provide adequate space for N_2_ molecules for adsorption as much as the model A and B. The steric effect of the methyl groups may be the main reason preventing the attachment of N_2_ molecules to the framework compared to the planar configurations of aromatic structures.^37^

**Fig. 6:**
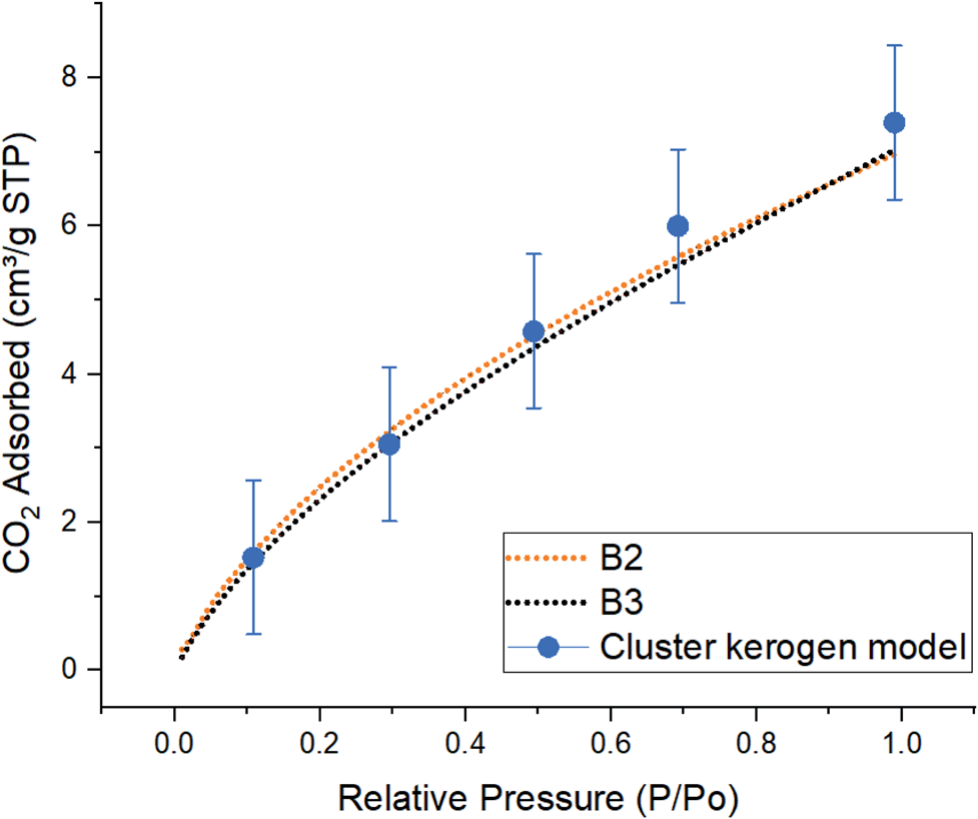
Comparison of simulated excess CO_2_ adsorption isotherms between the cluster kerogen model, blue dots, and the experimental samples B2, orange, and B3, black, at 273 K.

Since the overall results of N_2_ adsorption on the three models were close to the experiment, we clustered the three models for CO_2_ gas adsorption and diffusion simulations. Packmol package was utilized to place one of each kerogen models (A, B, and C) in two sides of a feed compartment with the size of around 16 × 57 × 40 angstrom, as shown in Fig. 1. These two systems were then allowed to come to relaxation by running a 1 ps NVT molecular dynamics simulations. The final average density of the kerogen models compartment is 0.922 g cm^−3^. Since the three kerogen models were simply adhered to one another and clustered, packing them in different modes was not considered. In this system, gaseous fluids would diffuse to two different surfaces of the clustered kerogen model. Unlike N_2_ gas adsorption experimental conditions, CO_2_ gas adsorption experiment, and accordingly theoretical simulations, were performed under a series of varying pressure values at 273 K (Fig. 6).

The result of CO_2_ gas adsorption isotherms of the samples B2 and B3 show a nearly linear relationship of gas adsorption with respect to the pressure. The cluster model very closely follows this behavior and only slightly deviates at higher pressure. At lower pressure of 10, 30, and 50 kPa, the cluster model shows a total amount of adsorbed CO_2_ molecules of 1.53, 3.05, and 4.58 cm^3^ g^−1^ STP, respectively. These are very close to those of experimental samples B2 (1.59, 3.22, and 4.52 cm^3^ g^−1^ STP), and B3 (1.45, 3.05, and 4.35 cm^3^ g^−1^ STP). The small discrepancy, around 5%, between the cluster model and samples B2 and B3 occurs when the simulation and experimental pressures reach 70 kPa. This phenomenon can be explained due to the increase in chemical potential in the smaller pores. As the pore radius decreases, the overlapping potentials from the strong pore wall–wall interactions and the strong CO_2_–wall interactions would lead to higher amounts of CO_2_ molecules to get adsorbed in smaller pores compared to the larger ones.^38,39^ Since the model hosts ultra-micro pores (0.3 nm to 0.7 nm) and larger number of CO_2_ molecules are placed in a fixed system at higher pressures (larger number of CO_2_ molecules in GCMC/MD simulation), it is observed that higher quantities of CO_2_ are adsorbed on the pore surfaces. This is in contrast with how samples B2 and B3 that both contain meso (less than 3–5 nm) and ultra-micro pores performed. The results proclaim that the pore structure plays an important role in adsorption mechanisms as a function of pressure.

**Fig. 7:**
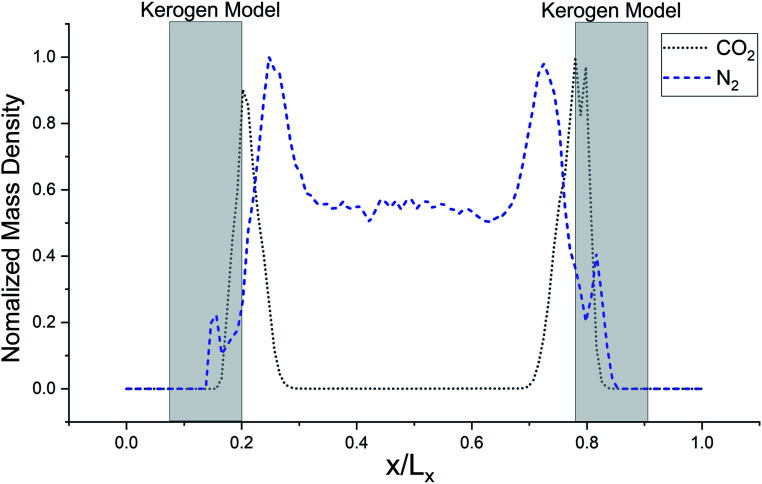
Normalized mass density profile of CO_2_ from clustered Bakken kerogen models at 100 kPa and 273 K, and of N_2_ from kerogen model A at 100 kPa and 77 K from GCMC/MD simulation. This density profile shows that CO_2_ and N_2_ molecules are crowded near Bakken models at one million time steps.

The simulated mass density profile in Fig. 7 shows that CO_2_ and N_2_ molecules have migrated to the kerogen model during the process and penetrated to the sub-surface levels of the model as well as being adsorbed on the surface. This simulation confirms that the interaction between gas (CO_2_ and N_2_) molecules and kerogen molecular models is strong enough to capture the molecules on or inside the models. Because the internal density of the model is irregular and highly densed sub-surfaces are existed (Fig. 3), the gas molecules could be captured into these densed areas inside the kerogen model. In particular, CO_2_ molecules show a much stronger interaction than N_2_ such that a considerable number of CO_2_ molecules penetrate to the sub-surface levels of the kerogen model. N_2_ molecules, on the other hand, are mostly diffused in the bulk region with a smaller number of molecules detained on the surface of the kerogen model. These results demonstrate that kerogen can be used as a porous filter for optimal separation of CO_2_ and N_2_ gas molecules.

## Conclusion

In this work, we reported a molecular model for amorphous organic matter (kerogen) built based on experimental constraints. The numerical analysis of the kerogen by the methods ^13^C-NMR, XPS, and XANES was used to determine the chemical composition and structure of three different models. GAFF parameters combined with partial charges computed *via* quantum mechanics calculations were used to build a more realistic model. GCMC and MD simulations were run to compute N_2_ and CO_2_ gas adsorption isotherms on the model and were compared to our experimental results. N_2_ gas adsorption behavior in the three kerogen model systems was in very good agreement with experimental results in similar conditions, 100 kPa and 77 K. Adsorption of CO_2_ molecules on a clustered model also shows similar adsorption isotherm behavior overall. Based on the simulation results we uncovered, the kerogen model seems to have a stronger interaction with CO_2_ molecules than N_2_ molecules such that CO_2_ molecules are not only adsorbed on the surface but also penetrate to the sub-surface level of the model.

## Conflicts of interest

There are no conflicts to declare.

## Supplementary Material
